# Asymptomatic Pulmonary Fibrosis Associated With Ipsilateral Proximal Interruption of a Pulmonary Artery (PIPA)

**DOI:** 10.7759/cureus.72572

**Published:** 2024-10-28

**Authors:** Jinyong Bae

**Affiliations:** 1 Radiology, Brooke Army Medical Center, San Antonio, USA

**Keywords:** absence of pulmonary artery, idiopathic pulmonary fibrosis, lung volume loss, proximal interruption of pulmonary artery, pulmonary fibrosis, pulmonary hypertension

## Abstract

Pulmonary fibrosis is a chronic condition typically affecting both lungs; however, cases of unilateral pulmonary fibrosis are exceedingly rare and often result from specific unilateral inflammatory conditions like radiation pneumonitis or infection. An even rarer occurrence is the unilateral proximal interruption of a pulmonary artery (PIPA), a developmental anomaly resulting from the failed connection of the sixth aortic arch to the pulmonary trunk. This condition can manifest alone or alongside other cardiac abnormalities. There are limited reports of pulmonary fibrosis associated with PIPA.

In this case, a 33-year-old male with chronic mild asthma presented with bilateral shoulder pain. Initial radiographs showed reticular opacities and volume loss in the right lung with a rightward mediastinal shift, suggesting possible fibrosis. Further investigations with chest Computed Tomography (CT) and CT angiogram confirmed right-sided pulmonary fibrosis and the absence of the right pulmonary artery, with no other significant cardiopulmonary symptoms reported. This case highlights the complexity of diagnosing and managing rare unilateral pulmonary conditions.

## Introduction

Idiopathic pulmonary fibrosis is a debilitating lung disease, typically presenting as a progressive, chronic disease affecting both lungs. However, unilateral pulmonary fibrosis is an uncommon condition linked to various chronic unilateral inflammatory issues such as radiation pneumonitis [1,2], infection [3,4], pulmonary vein thrombosis [5], and single-lung ventilation [6]. Another rare condition, unilateral proximal interruption of a pulmonary artery (PIPA), arises from the failure of the sixth aortic arch to connect with the pulmonary trunk [7,8]. This defect, which may affect either the right or left side, often co-occurs with congenital heart anomalies like tetralogy of Fallot or septal defects [9,10], although it can also present independently. There are only a few documented cases of pulmonary fibrosis associated with PIPA [11]. 

## Case presentation

**Figure 1:**
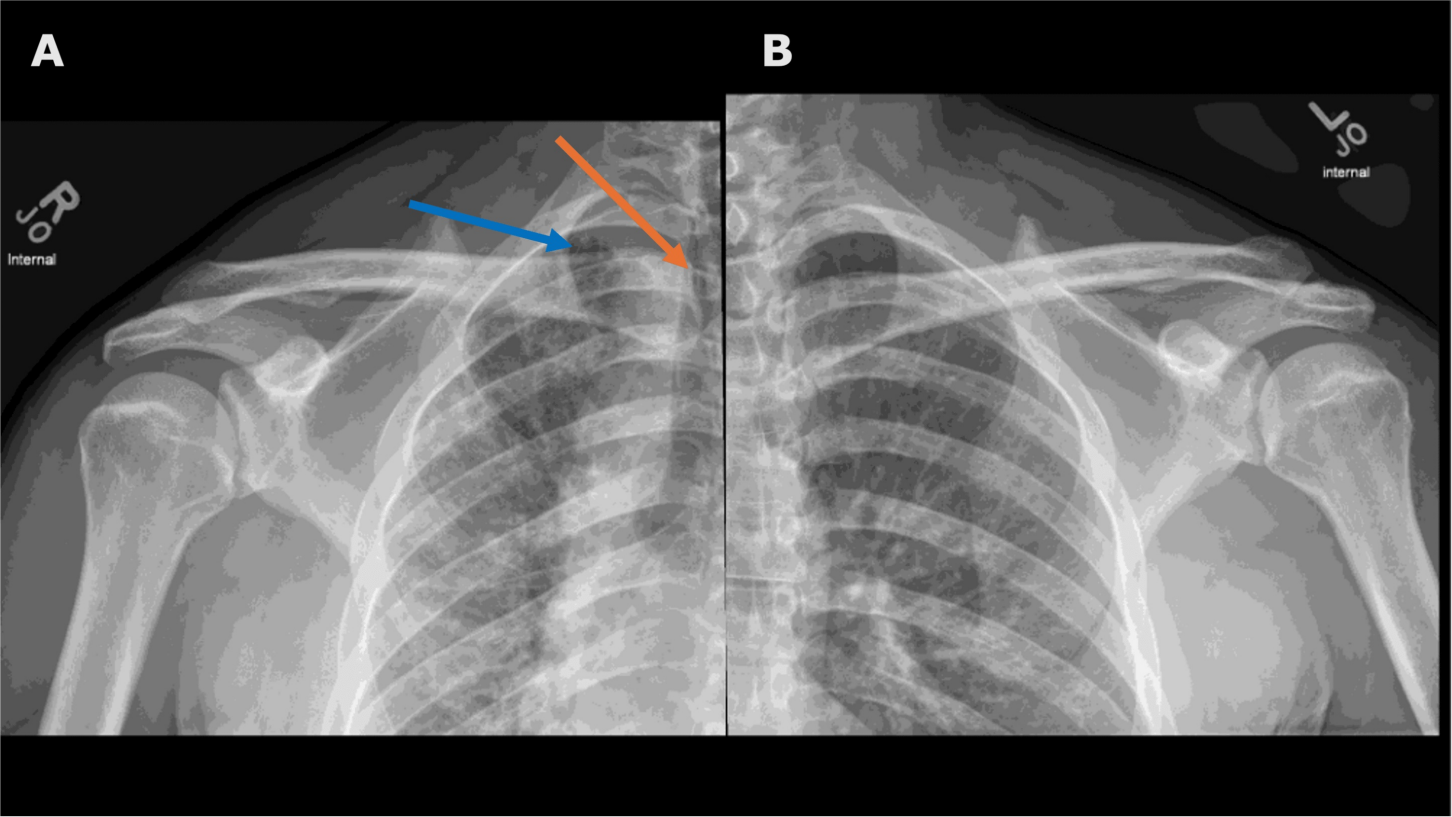
Bilateral shoulder X-rays evaluating causes for shoulder pain. The bilateral shoulder radiographs reveal a decrease in volume in the right lung, evidenced by a shift in the mediastinum and trachea (orange arrow). Additionally, there is a noticeable enhancement of the interstitial patterns and reticulation in the right lung (left image, A), especially in the upper lung (blue arrow). The left lung appears normal on the radiograph (right image, B). Bilateral shoulder pain was diagnosed as mild to moderate osteoarthritis.

A 33-year-old active-duty military male presented to a primary clinic due to bilateral shoulder pain. He has history of mild asthma but denied significant history of smoking, occupational risk, recurrent pneumonia or hemoptysis, or current cardiopulmonary symptoms like cough, shortness of breath, chest pain, or palpitations. Bilateral shoulder radiographs were taken for his shoulder pain (Figure 1). These images showed reticular opacities and volume loss in the right lung, along with a mediastinal shift to the right side, suggesting possible scarring or fibrosis. 

**Figure 2:**
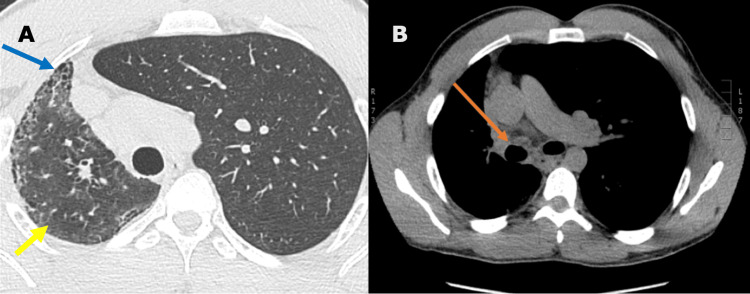
Pulmonary fibrosis of the right lung and absence of right pulmonary artery In the non-contrast CT chest of the axial lung window (A), there are mild peripheral honeycombing (blue arrow), reticulations, and subpleural ground-glass opacities (yellow arrow), which are likely related to the absence of the right pulmonary artery. The axial soft tissue window (B) further confirms the absence of the right pulmonary artery (orange arrow). Additionally, there is a rightward shift of the mediastinum, moderate volume loss in the right lung, and compensatory expansion of the left lung.

**Figure 3:**
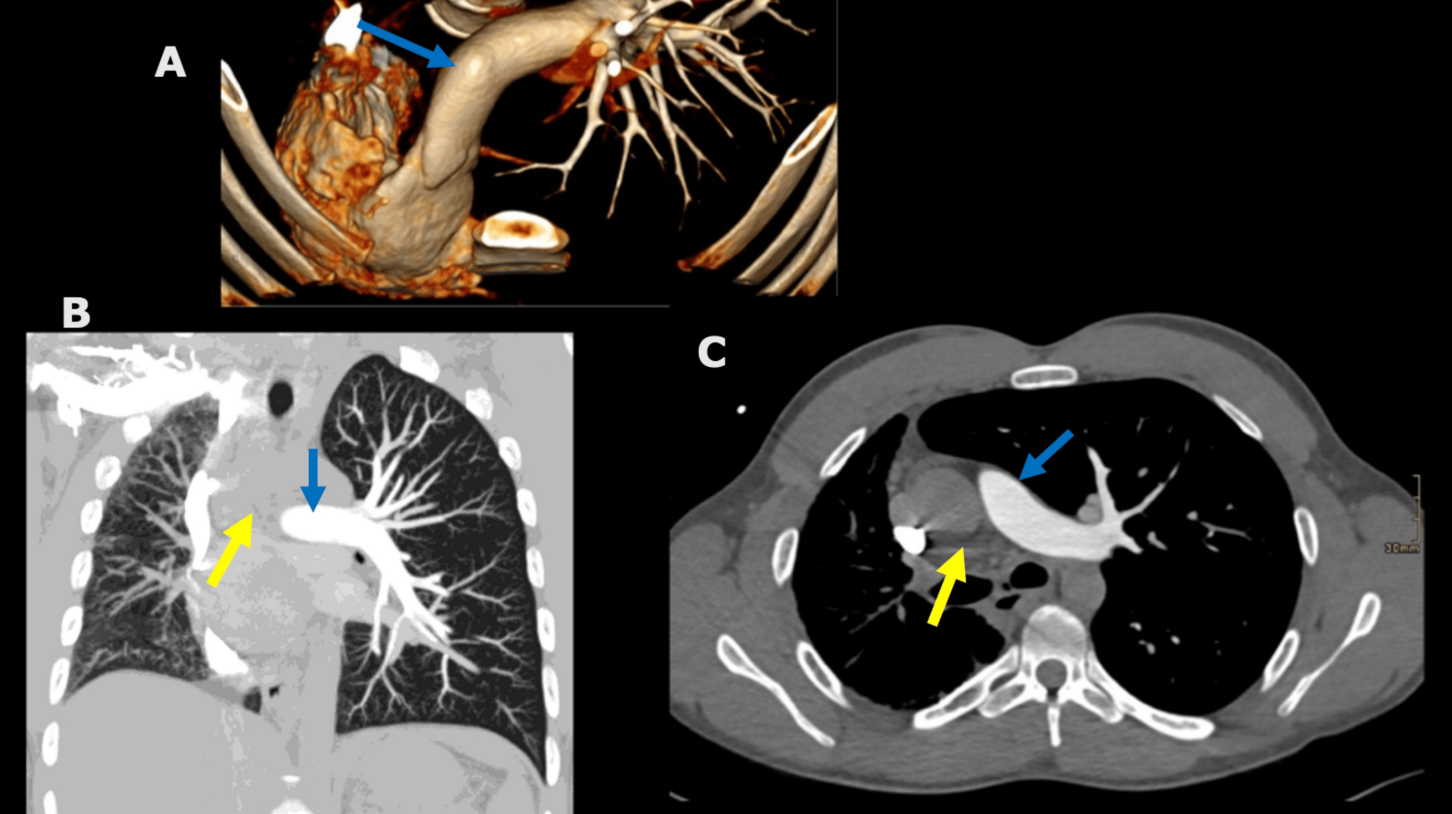
Absence of right pulmonary artery Axial 3-dimensional volume rendering (A), coronal (B), and axial (C) images of CT chest angiogram. Normal appearing left pulmonary artery (blue arrow) and missing right pulmonary artery (yellow arrow) are demonstrated.

Further evaluation through chest CT and CT chest angiogram confirmed right-sided pulmonary fibrosis and volume loss, a mediastinal shift from left to right, and an absence of the right pulmonary artery (Figures 2, 3). The findings prompted a referral to a pulmonologist who planned further investigations for the PIPA and associated pulmonary fibrosis. Unfortunately, the patient failed to attend his scheduled tests and was subsequently lost to follow-up. 

## Discussion

According to a review article, the median age for patients diagnosed with PIPA is 14 years [8]. About 13% of these cases are asymptomatic, but the most common symptoms include pulmonary hypertension (44%), dyspnea or limited exercise tolerance (40%), frequent pulmonary infections (37%), and hemoptysis (20%) [8]. 

Currently, there is no universally accepted approach for diagnosing and treating PIPA. However, plain radiographs and non-contrast chest CT scans are commonly used diagnostic tools.

One reported case describes a 30-year-old female with right-sided pulmonary fibrosis and absence of the right pulmonary artery, presenting with mild chronic asthma and shortness of breath [11]. This case closely resembles ours, except for symptoms such as shortness of breath. 

Asthma is often marked by airway remodeling- changes in the airway wall, including epithelial damage, smooth muscle hypertrophy and hyperplasia, collagen buildup, subepithelial membrane thickening, and fibrosis [12]. While fibrosis is common in long-term severe asthma, it is rare in mild asthma, especially among younger patients [12]. In PIPA, fibrosis mechanisms are not entirely clear but may involve inflammation-driven reactive oxygen species (ROS) [13]. 

Isolated unilateral PIPA in adults can cause significant hemodynamic disturbances in pulmonary circulation, leading to recurrent hemoptysis, cystic lung lesions, and pulmonary vascular malformations, generally resulting in a poor long-term prognosis [10]. Early detection, however, can enable long-term therapeutic interventions that significantly enhance quality of life [10]. 

Patients with conditions that are associated with pulmonary hypertension, such as obstructive sleep apnea, congenital heart defects, autoimmune diseases like scleroderma and lupus, as well as chronic liver disease, may require close monitoring. Additionally, avoiding high altitudes or pregnancy may help mitigate exacerbations of these conditions. 

Treatment options for PIPA range from optimized medical management of symptoms to surgical intervention. However, surgical treatment is generally recommended only for patients who exhibit severe symptoms, such as cardiac failure and significant pulmonary hypertension [14]. 

## Conclusions

The absence of the right pulmonary artery was incidentally discovered alongside right-sided pulmonary fibrosis in an asymptomatic young male patient. Possible mechanism for fibrosis in PIPA cases include chronic inflammation from asthma or exposure to ROS. Early diagnosis allows timely care and informs patients of the importance of seeking prompt medical attention if symptoms appear. 

## References

[1] Mehta V (2005). Radiation pneumonitis and pulmonary fibrosis in non-small-cell lung cancer: pulmonary function, prediction, and prevention. Int J Radiat Oncol Biol Phys.

[2] Karlsen J, Tandstad T, Sowa P, Salvesen Ø, Stenehjem JS, Lundgren S, Reidunsdatter RJ (2021). Pneumonitis and fibrosis after breast cancer radiotherapy: occurrence and treatment-related predictors. Acta Oncol.

[3] Ellson CD, Dunmore R, Hogaboam CM, Sleeman MA, Murray LA (2014). Danger-associated molecular patterns and danger signals in idiopathic pulmonary fibrosis. Am J Respir Cell Mol Biol.

[4] Azadeh N, Limper AH, Carmona EM, Ryu JH (2017). The role of infection in interstitial lung diseases: a review. Chest.

[5] Cavaco RA, Kaul S, Chapman T, Casaretti R, Philips B, Rhodes A, Grounds MR (2009). Idiopathic pulmonary fibrosis associated with pulmonary vein thrombosis: a case report. Cases J.

[6] Anand SH, Jasper A, Mani SE, Joseph E, Mathai J (2015). Proximal interruption of the pulmonary artery: a case series. J Clin Diagn Res.

[7] Kimura T, Mizutani T (2001). [Unilateral pulmonary fibrosis following ipsilateral single-lung ventilation and anesthesia]. Masui.

[8] Ten Harkel AD, Blom NA, Ottenkamp J (2002). Isolated unilateral absence of a pulmonary artery: a case report and review of the literature. Chest.

[9] Pool PE, Vogel JH, Blount Jr SG (1962). Congenital unilateral absence of a pulmonary artery. The importance of flow in pulmonary hypertension. Am J Cardiol.

[10] Jariwala P, Maturu VN, Christopher J, Jadhav KP (2021). Congenital isolated unilateral agenesis of pulmonary arteries in adults: case series and review. Indian J Thorac Cardiovasc Surg.

[11] Sumdani H, Shahbuddin Z, Farhataziz N, Barkley JM (2019). Unilateral pulmonary fibrosis due to absence of right pulmonary artery. Cureus.

[12] Savin IA, Zenkova MA, Sen'kova AV (2023). Bronchial asthma, airway remodeling and lung fibrosis as successive steps of one process. Int J Mol Sci.

[13] Morry J, Ngamcherdtrakul W, Yantasee W (2017). Oxidative stress in cancer and fibrosis: opportunity for therapeutic intervention with antioxidant compounds, enzymes, and nanoparticles. Redox Biol.

[14] Toews WH, Pappas G (1983). Surgical management of absent right pulmonary artery with associated pulmonary hypertension. Chest.

